# Comparative study of the impact of dietary supplementation with different types of CpG oligodeoxynucleotides (CpG ODNs) on enhancing intestinal microbiota diversity, antioxidant capacity, and immune-related gene expression profiles in Pacific white shrimp (*Litopenaeus vannamei*)

**DOI:** 10.3389/fimmu.2023.1190590

**Published:** 2023-04-27

**Authors:** Feng Hu, Yan Wang, Jingjie Hu, Zhenmin Bao, Mengqiang Wang

**Affiliations:** ^1^ MOE Key Laboratory of Marine Genetics and Breeding, and Key Laboratory of Tropical Aquatic Germplasm of Hainan Province of Sanya Oceanographic Institute, Ocean University of China, Qingdao, China; ^2^ Hainan Yazhou Bay Seed Laboratory, Sanya, China; ^3^ Laboratory for Marine Fisheries Science and Food Production Processes, and Center for Marine Molecular Biotechnology, Laoshan Laboratory, Qingdao, China

**Keywords:** Litopenaeus vannamei, CpG oligodeoxynucleotides, intestinal microbiota, innate immunity, immune modulation

## Abstract

The CpG oligodeoxynucleotides (CpG ODNs) reportedly possess the capacity to strengthen immunity in mammals. This experiment was conducted to evaluate the impact of dietary supplementation with 17 types of CpG ODNs on intestinal microbiota diversity, antioxidant capacity, and immune-related gene expression profiles of the shrimp *Litopenaeus vannamei*. Diets including 50 mg kg^-1^ CpG ODNs wrapped in egg whites were prepared and divided into 17 different groups, with 2 control groups (normal feed and feed with egg whites). These CpG ODNs supplemented diets and the control diets were fed to *L. vannamei* (5.15 ± 0.54 g) three times daily at 5%-8% shrimp body weight for three weeks. The results of consecutive detection of intestinal microbiota by 16S rDNA sequencing indicated that 11 of the 17 types of CpG ODNs significantly enhanced intestinal microbiota diversity, increased the populations of several probiotic bacteria, and activated possible mechanisms relevant to diseases. The immune-related genes expression and antioxidant capacity in hepatopancreas further demonstrated that the 11 types of CpG ODNs effectively improved the innate immunity of shrimp. Additionally, histology results showed that the CpG ODNs in the experiment did not damage the tissue structure of hepatopancreas. The results suggest that CpG ODNs could be used as a trace supplement to improve the intestinal health and immunity of shrimp.

## 1 Introduction

Due to the rapid growth rate and the ability to tolerate wide range of temperatures and salinities, the Pacific white shrimp, *Litopenaeus vannamei*, has become one of the most profitable varieties in crustacean aquaculture (1, 2). However, the extended shrimp farming industry has sustained declining disease resistance, metabolic disorder, and frequent outbreaks of serious diseases, including viral an bacterial diseases, resulting in huge financial losses. Among all the shrimp bacterial diseases, vibriosis, which causes significant production losses, is of particular concern (3). Vaccines, such as subunit and recombinant treatments, are too costly for practical production on account of the individual size and farming scale of shrimp (4), while drugs such antibiotics carry the risk of excessive abuse, which can lead to a variety of drug-resistant bacteria, and disordered immune function, intestinal microbial flora imbalance, and even drug residues of aquatic animal (5). Therefore, it is necessary to develop dietary additives that can be used as immunoenhancement to enhance the innate immune capacity of shrimp (6).

CpG Oligonucleotides (CpG-ODNs) are synthetic short DNA with a CpG motif, which mimic bacterial genomic DNA and are significant potent activators of adaptive and innate immunity (7). CpG-ODNs are generally divided into four classes (A, B, C, and P) according to their structural differences, and each class of CpG-ODNs exhibits different immunostimulatory and immunomodulation effects (7). The A-class CpG-ODNs participate in the humoral immune response (8), the B-class CpG-ODNs relate to the cellular immune response (9), the C-class CpG-ODNs participate in both humoral and cellular immune responses (10), while the P-class CpG-ODNs affect hematology and plasma cytokines (11). Meanwhile, N-class CpG ODNs is a type of CpG-ODNs that cannot induce any immune stimulation on account of the backbone and the number, nature and spacing of the CpG motifs. For example, CpG 1720, CpG 2137 and CpG 2243 did not enhance the immunity of olive flounder (*Paralichthys olivaceus*) (12) and rainbow trout (*Oncorhynchus mykiss*) (13), respectively. CpG ODNs with similarly bacterial DNA motifs can typically initiate some innate immune responses by the recognition of molecules bearing conserved motifs, termed pathogen-associated (PAMPs), *via* pattern recognition receptors (PRRs) (14). It has been reported that CpG-ODNs are an immune response modifier in mammals (15). There are also slight differences in various animal models in terms of the immune stimulation effects caused by CpG ODNs. In murine and primate models, CpG-ODNs target Toll-like receptor (TLR) to trigger an immunostimulatory and immunomodulation cascade or stimulate an innate immune response, thereby contributing to the clearance of bacteria, parasites, and viruses and reducing the severity and duration of infection (16). In aquatic organisms, CpG ODNs can induce high resistance against viral hemorrhagic septicemia virus infection and intensive expression levels of *Mx* and *ISG15* genes in *Paralichthys olivaceus* (12), produce remarkably high levels of O^2−^ and H_2_O_2_ on macrophages and high anti-bactericidal activities in grass carp (17), and induce proliferation of spleen and head kidney cells, and peripheral blood leucocytes, from rainbow trout (18). The previous study verified that oral administration of CpG ODNs reduced the death rate of shrimps exposed to WSSV (19). However, it is unclear how CpG ODNs affect the intestines of shrimp. Therefore, it is necessary to investigate the mechanism of CpG-ODNs *via* intestinal absorption and recognition when stimulating innate immune response in *L. vannamei*.

The intestine is the main organ of shrimp digestion, absorption, and nutrient metabolism, as well as a significant component of its defense against pathogen invasion, which is mainly realized through the intestinal mucosal barrier. Due to the absence of an adaptive immune system, the intestinal mucosal barrier of *L. vannamei* plays a significant role in its innate immune system, which mainly consists of a mechanical, a chemical, and a microbial barrier (5, 20). The microbial barrier refers to the combination and adhesion of intestinal flora with intestinal mucosa, forming a microecosystem composed of membrane flora with certain regularity, and interdependent and interacting with the microspatial structure of the host. Many studies have shown that intestinal microbial community structure is closely related to the immunity and disease resistance of shrimp (21, 22). Therefore, the intestinal microbial community is a potential target for immune regulation of *L. vannamei*, and it is expected to achieve effective prevention and control of related diseases through immune regulation of intestinal microbiota structure.

In this study, 17 types of CpG ODNs (classified into A, B, C, P, N) were synthesized and added to shrimp feed. The variation of intestinal flora in shrimp was testified by 16S rDNA sequencing after continuous feeding to investigate the effects of each CpG ODN on the intestinal microbiota structure. The experiment of immune-related genes expression and antioxidant capacity in hepatopancreas was conducted to study the effects of CpG ODNs on nonspecific immune parameters. Additionally, hepatopancreas histomorphology verified whether CpG ODNs exert a negative effect on the vital organs of shrimp. These results provide novel insights into the immunoenhancement of CpG ODNs to improve shrimp immunity.

## 2 Material and methods

### 2.1 Preparation of CpG ODNs

Seventeen types of CpG ODNs (**Table S1**), which were efficiently used in mammalian and aquatic animals, were synthesized, and inserted into plasmid pUC57 with 10 copies in series to form a series of CpG-rich DNA fragments with tandem CpG ODNs. Plasmids pUC57-CpG were massively extracted from *Escherichia coli* using a modified alkaline lysis method (23), then stored at -80 °C until further use.

### 2.2 Diets preparation

The dietary composition of shrimps is shown in **Table 1**. A basal diet was purchased from Guangdong Zhengge Aquatic Science and Technology Co., Ltd., pulverized with a grinder, and sieved using a 300-mesh size nylon sieve. Different kinds of CpG ODNs dissolved in small amount of egg white were added to basal diet pulverized powder and stirred equably, then 40% water was added until stiff doughs containing 50 mg kg^-1^ CpG ODNs were obtained. The dough was pelletized by double knife noodle pressing machine (Weidi, China) with diameters of 1.0 mm. Basal diet and basal diet with small amount of egg white (about 5 g kg^-1^), made according to the same method, were set as control groups. The details of groups and samples are shown in **Table S2**. All diets were placed in a fume hood to air dry to about 10% moisture and stored at -20 °C in a freezer for further use.

**Table 1:** Formulation and proximate composition of experimental diets (air dry basis, g/kg).

Ingredients	Composition (g/kg)
Fish meal	300
Wheat flour	70
Soybean meal	80
Shrimp meal	100
Yeast extract	100
Fish oil	20
Soybean lecithin	40
Squid meal	270
Vitamin premix	10
Mineral premix	10
Total	1000
Proximate composition	
Crude protein	421.6
Crude fat	76.5
Ash	132.5
Moisture	93.1
Total phosphorus	15.8

### 2.3 Animal management

The feeding experiment was conducted in Renhai Aquatic Science Technology Co., Ltd., Hainan Province, China. Healthy shrimps with an average initial body weight of 5.15 ± 0.54 g were acclimatized for 7 days prior to the experiment, in filtered aerated seawater with 24 ± 1 °C in temperature and 10 ± 1 ppt in salinity. A total of 1140 *L. vannamei* shrimps were randomly separated into nineteen 1200 L water tanks containing 800 L of sea water (randomly assigned 60 tails per tank). The shrimps were fed at 08:00, 14:00, and 20:00 each day. The initial ratio was 5-8% of their body weight, and the feeding experiment lasted for 14 days.

### 2.4 Sample collection

The whole hepatopancreas samples from three shrimp per tank at 0-, 7- and 14-days after feeding were collected, and immediately placed into liquid nitrogen to await further analysis of enzyme activity and gene expression profiles. The hepatopancreas tissue samples from three shrimp per tank at 14 days post feeding were pooled and placed in 4% paraformaldehyde solution for further histological experiments. For each tank, completed intestines from three shrimps at 0-, 7- and 14-days post feeding were dissected and immediately placed in liquid nitrogen until further analysis of intestinal microbiota.

### 2.5 Total RNA isolation and cDNA synthesis

Total RNA from pulverized *L. vannamei* hepatopancreas tissues was extracted using TRIzol Reagent (ThermoFisher, Carlsbad, CA, USA) and completely digested with DNase I (TaKaRa, Shiga, Japan) to remove potential genomic DNA contamination. The concentration and purity of obtained RNA were determined using NanoDrop One (ThermoFisher, Waltham, MA, USA), and its integrity was further verified by agarose gel electrophoresis analysis. The first-strand cDNA was synthesized using Reverse Transcriptase M-MLV (RNase H^-^) Kit (Takara, Shiga, Japan) with random primers using 1 μg total purified RNA.

### 2.6 Gene expression analysis

Six pairs of primers (shown in **Table 2**), including interleukins (*IL-1-β*, *IL-8*, *IL-10*), inflammatory cytokines (*TNF-α*), anti-lipopolysaccharide factor (*ALF*) and target of rapamycin (*TOR*), were selected to analyze the immune-related gene expression during a 14-day feeding period. The assays were performed on a Light Cycler 480 (Roche, Mannheim, Germany) with 10 μL of 2×SYBR qPCR Master Mix (Everbright, Suzhou, China), 0.4 μL of forward primer (10 μM), 0.4 μL of reverse primer (10 μM), 1 μL diluted cDNA template (20 ng/μL) and 8.2 μL ddH_2_O using the following experiment set: 95 °C for 5 min, and 95 °C for 15 s and 35 cycles, 60 °C for 45 s. The *β-actin* gene was employed as an internal control (**Table 2**). All the qPCR reactions were repeated with two independent samples, and performed for three biological replicates. Relative mRNA expression was generated and calculated using 2^-ΔΔCt^ method (24).

**Table 2:** Primer sequences for qPCR.

Gene	Primer sequences (5’-3’)	Accession Number
*IL-1β*	F: AGAGTTTGGTGAAGAAGAGG	JQ692172
	R: TTATTGTGGTTACGCTGGA	
*IL-8*	F: GCACTGCCGCTGCATTAAG	DQ061114.1
	R: GCAGTGGGAGTTGGGAAGAA	
*IL-10*	F: AATCCCTTTGATTTTGCC	HQ388294
	R: GTGCCTTATCCTACAGTATGTG	
*TNF-α*	F: CGCTGCTGTCTGCTTCAC	HQ696609
	R: CCTGGTCCTGGTTCACTC	
*ALF*	F: CATTCGGCCTTGACTTCG	XM_027372864.1
	R: ATCCAGGACACCACATCCTG	
*TOR*	F: GCAGATCCTTGAGAAGACGC R: CTGACAGCCGCATTGAGGTA	XM_027365549.1
*β-Actin*	F: GGCTGTGCTGTCCCTGTA R: GGGCATAACCCTCGTAGAT	MN639305.1

### 2.7 Analysis of antioxidant index

Hepatopancreas samples from the shrimps at 0-, 7- and 14- days after feeding were used to detect the activities of antioxidant enzymes, including superoxide dismutase (SOD), catalase (CAT), glutathione peroxidase (GPX), and glutathione S-transferase (GST). Superoxide Dismutase (SOD) assay kit, Catalase (CAT) assay kit, Glutathione Peroxidase (GSH-PX) assay kit and Glutathione S-transferase (GSH-ST) assay kit (Jiancheng, Nanjing, China) were used for these assays according to the manufacturer’s protocols.

### 2.8 Histomorphology

The hepatopancreas tissue of *L. vannamei* at 14 days after feeding was immobilized in 4% paraformaldehyde fixative solution (Solarbio, Beijing, China) for 24 h and then dehydrated in various concentrations of alcohol (from 50% to 95%). The dehydrated tissues were then embedded in paraffin, and cut into thick slices with 4 μm in thick. The obtained tissues sections were stained with hematoxylin and eosin (H&E), then observed with a BX53M light microscope (Olympus, Tokyo, Japan).

### 2.9 Intestinal microbiota analysis

DNA from the intestines of *L. vannamei* shrimps after 0-, 7- and 14- days of feeding, respectively, was extracted using a commercial DNA extraction kit (Omega, Guangzhou, China). The V3-V4 region of microbial 16S rDNA was amplified obtain an about 420 bp amplified fragment. The Illumina NovaSeq 6000 platform were used to obtain 2×250 bp paired-end sequence data. Paired-end reads were assigned to samples, truncated, and then merged using FLASH 1.2.8 for 16S or PEAR 0.9.6 for ITS2 (25). Raw reads were processed under specific filtering conditions to obtain the high-quality clean reads using fqtrim 0.94. Chimeric sequences were detected and filtered using V search software 2.3.4 (26). After dereplication using Divisive Amplicon Denoising Algorithm (DADA2), feature table and feature sequence of amplicon sequence variants (ASVs) were obtained. Alpha diversity and beta diversity were calculated according to the QIIME2 process, and visualized by R script. The sequence alignment of species annotation was performed by Blast against SILVA and NT-16S database (27). Based on the obtained species abundance statistics, the differences between the experimental and control groups were obtained and analyzed. A Mann-Whitney U test was used for comparison of the differences between the two groups of samples with biological replicates, while Kruskal–Wallis’s test was applied to compare multiple groups with biologically replicated samples (*p*<0.05). PICRUSt2 software was employed to predicts functional abundance based on marker gene sequences (28). The function of intestinal flora with relatively high abundance was annotated by PICRUSt2 (https://github.com/picrust/picrust2) based on COG (Clusters of Orthologous Groups) database. The differences between experimental and control groups were emphasized. BugBase (29) was employed to predict the phenotype of an intestinal microbial sample from each group.

### 2.10 Statistical analysis

Significant differences among different groups were tested by one-way analysis of variance (ANOVA) using SPSS 26.0 (IBM, Armonk, NY, USA). All data are presented in the form of means ± standard error (n = 3). Duncan’s multiple-range test was applied for multiple comparisons of group means. The histograms were implemented using OriginPro 2022b (Northampton, Massachusetts, USA). The difference was considered significant at a *p* value < 0.05.

## 3 Results

### 3.1 The expression of immune-related genes in hepatopancreas

**Figure 1:**
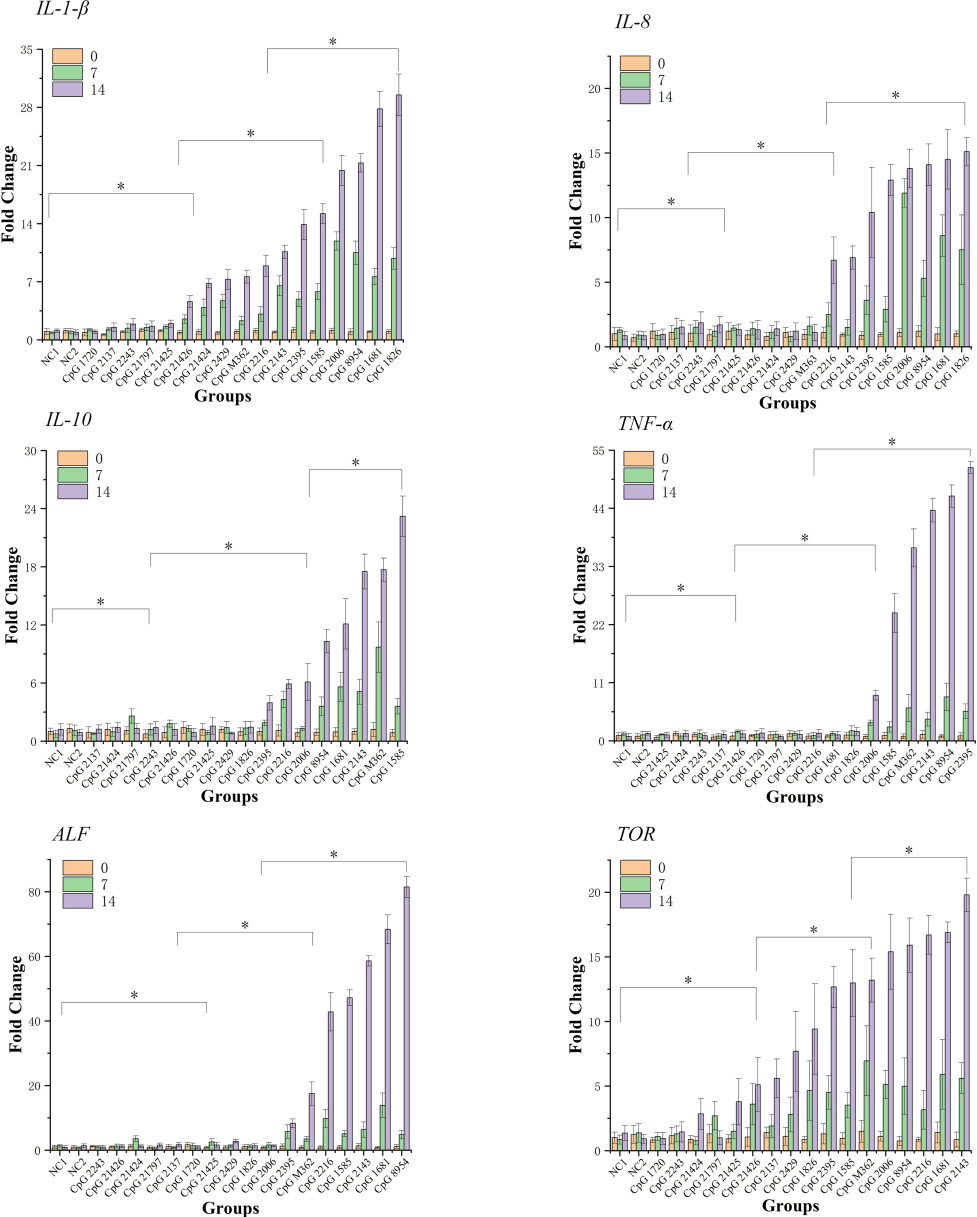
qPCR analysis of the expression of six immune-related genes detected in hepatopancreas of shrimps at 0-, 7- and 14-days. Data are means for three assays and presented as the means ± SD (*p* < 0.05). The symbol * indicates significant differences (p < 0.05).

As shown in **Figure 1**, after 14 days’ feeding, the expression of six immune-related genes exhibited amplitude and trends of different degrees of upregulation in most experimental groups, while some groups (CpG 1720, CpG 2137, CpG 21424, CpG 2243, CpG 21797, CpG 21425) consistent with the negative control groups did not reveal a stimulation effect for these genes. In the first seven days, at least 7 of 19 groups and at most 13 of 19 groups exhibited upregulation of all six detected genes. From seven to fourteen days, several groups, such as CpG 2395, CpG 1585, CpG 1681, CpG 8954, CpG 2143 and CpG M362, showed intense upregulation, while other groups exhibiting a tiny amount of upregulation (*p*<0.05).

### 3.2 The antioxidant capacity in hepatopancreas

**Figure 2:**
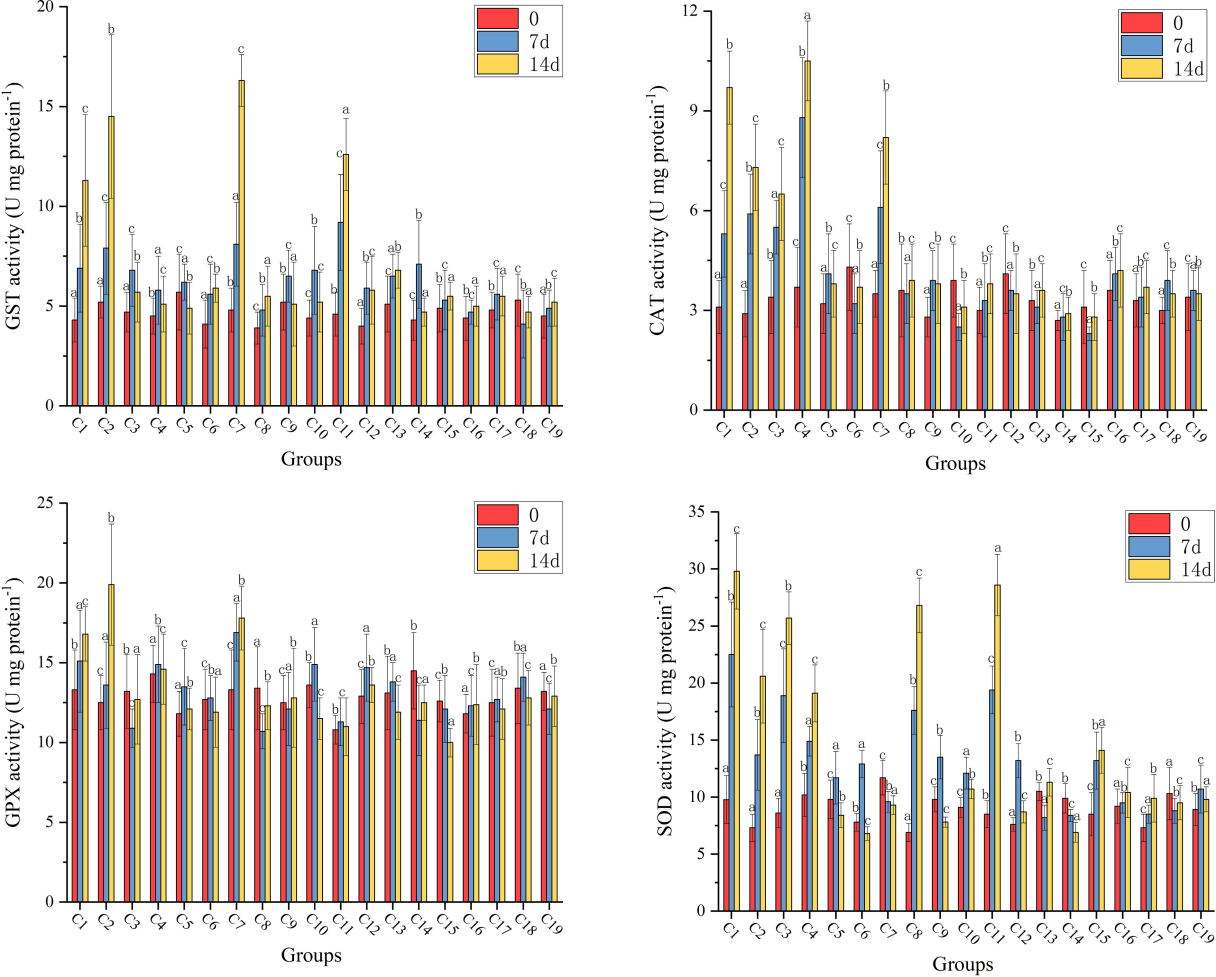
The results of the four antioxidant enzymes activity (GST, CAT, GPX and SOD) analysis in hepatopancreas of shrimps at 0-, 7- and 14-days. Data are means for three assays and presented as the means ± SD, different letters indicate significant differences (*p* < 0.05).

**Figure 2** illustrates the results of the four antioxidant enzymes activity (GST, CAT, GPX and SOD) analysis in hepatopancreas after 14 days’ feeding. Compared with the two negative control groups, of which the four antioxidant enzymes activities did not show any significant changes over 14 days, the C1, C2, C7, and C11 experimental groups exhibited significantly higher GST activity at 14 days than at 0 days (*p*<0.05); the C1, C2, C3, C4, and C7 experimental groups indicated significantly higher CAT activity at 14 days than at 0 days (*p*<0.05); the C1, C2, and C7 experimental groups showed significantly higher GPX activity at 14 days than at 0 days (*p*<0.05); and the C1, C2, C3, C4, C8, C9, and C15 experimental groups exhibited significantly higher SOD activity at 14 days than at 0 days (*p*<0.05). Additionally, no significant changes in antioxidant enzymes activity of the residual experimental groups were observed.

### 3.3 Hepatopancreas histomorphology

**Figure 3:**
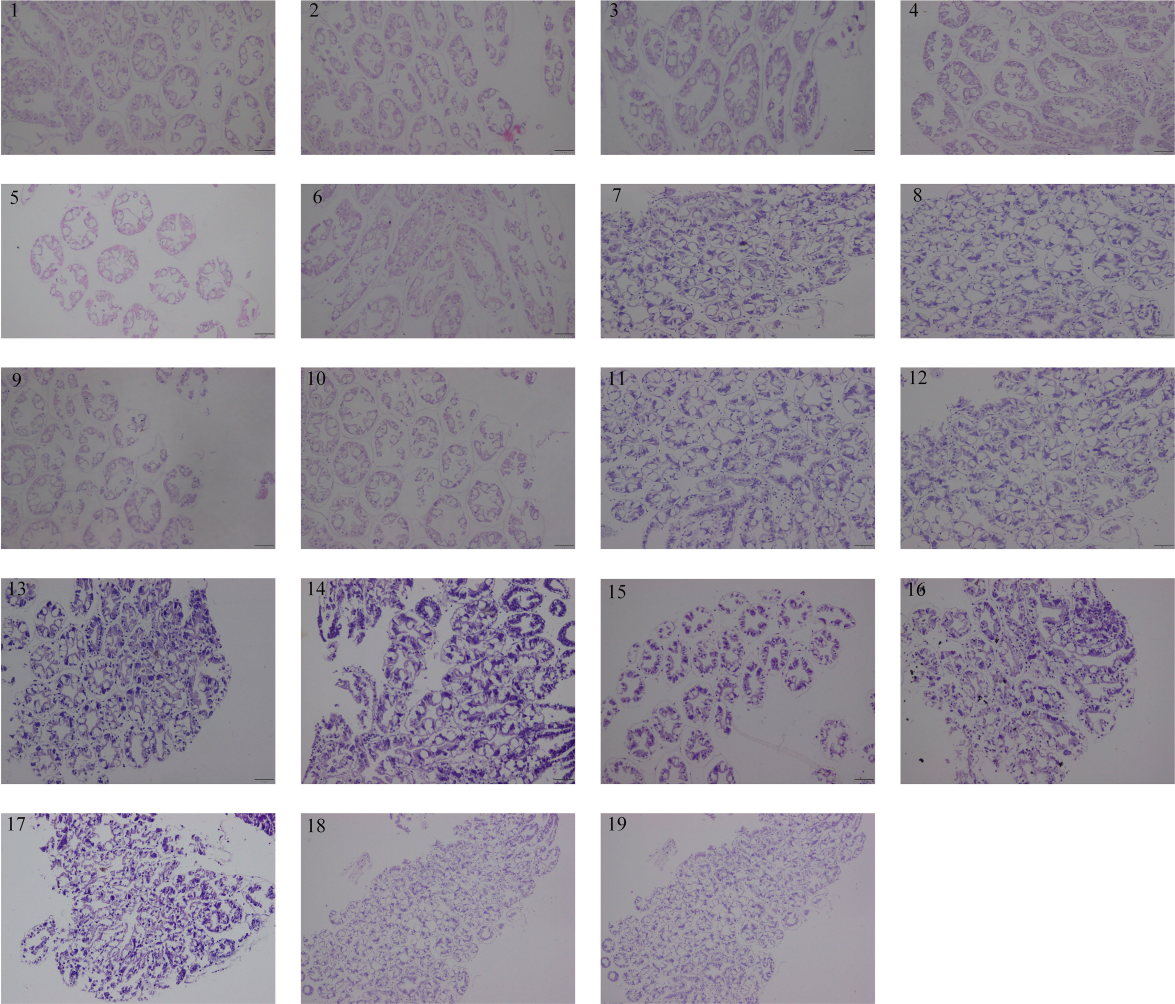
Hepatopancreas histomorphology of shrimps from each group at 14 days. All sections were stained with H&E. Scale bar 100 µm. 1. CpG ODN 1826, 2. CpG ODN 1681, 3. CpG ODN 2006, 4. CpG ODN M362, 5. CpG ODN 2137, 6. CpG ODN 8954, 7. CpG ODN 1585, 8. CpG ODN 2143, 9. CpG ODN 2395, 10. CpG ODN 2216, 11. CpG ODN 23617, 12. CpG ODN 2429, 13. CpG ODN 21424, 14. CpG ODN 21425, 15. CpG ODN 21426, 16. CpG ODN 2243, 17. CpG ODN 1720, 18. Negative control 1, 19. Negative control 2.

Hepatopancreas of Pacific white shrimp stained by H&E from each group were presented in **Figure 3**. The hepatopancreas tissue morphology of *L. vannamei* of all groups was characterized as normal. There were no hepatopancreas morphological alterations observed among all the experimental treatments. These results revealed that trace CpG ODNs in the diet had no obvious effect on the health status of the hepatopancreas tissue.

### 3.4 Alpha diversity analysis

A total of 118,414,123 high-quality sequences were obtained from 171 experimental samples of the 19 groups. All the raw sequencing data were submitted to NCBI (PRJNA877472). ASV feature sequences were denoised by QIIME2, and randomly selected to calculate the values of alpha diversity (**Supplementary: Figure S1**). The community coverage of each sample was greater than 99% (**Supplementary: Table S3**), indicating that the identified sequence in the present research can represent most of the microorganism in each sample. Both Shannon and Simpson indices reflect the high community diversity, the Chao indices present high community richness, and the pielou-e indices exhibit Shannon’s evenness (**Supplementary: Table S3**). The results indicated that after two weeks of feeding, the alpha diversity of intestinal microbiota in the groups C1, C2, C3, C4, C6, C7, C8, C9, C10, C11, C12, C15 and C16 constantly increased, and was significantly higher than those in the two control groups, suggesting that the intestinal microbial abundances of shrimps in those groups were strengthened.

### 3.5 Beta diversity analysis

**Figure 4:**
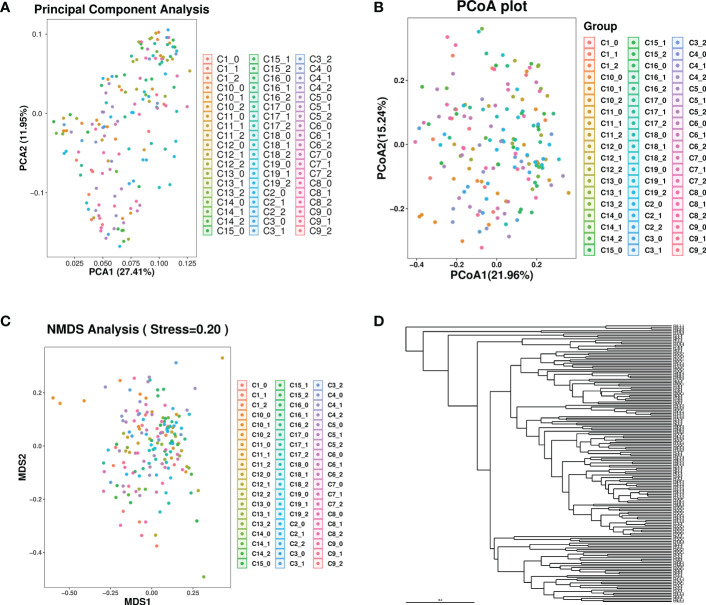
The results of beta diversity from each group at 0, 7 and 14 days. **(A)** Principal component analysis (PCA), **(B)** Principal coordinates analysis (PCoA), **(C)** Nonmetric multidimensional scaling (NMDS), **(D)** Unweighted pair group method with arithmetic mean (UPGMA).

Together, beta and alpha diversity constitute the overall diversity or biological heterogeneity of certain microbiological communities. Principal component analysis (PCA), principal coordinates analysis (PCoA), an unweighted pair group method with arithmetic mean (UPGMA), and nonmetric multidimensional scaling (NMDS) were used to observe the differences between samples (**Figure 4**). The results indicated that the species diversity of intestinal microbial communities varied significantly in three samples (0-, 7- and 14-days) in the groups of C1, C2, C3, C4, C6, C7, C8, C9, C10, C11 and C15. This was due to the function of different CpG ODNs rather than other environmental factors.

### 3.6 The microbial community composition in the intestine

**Figure 5:**
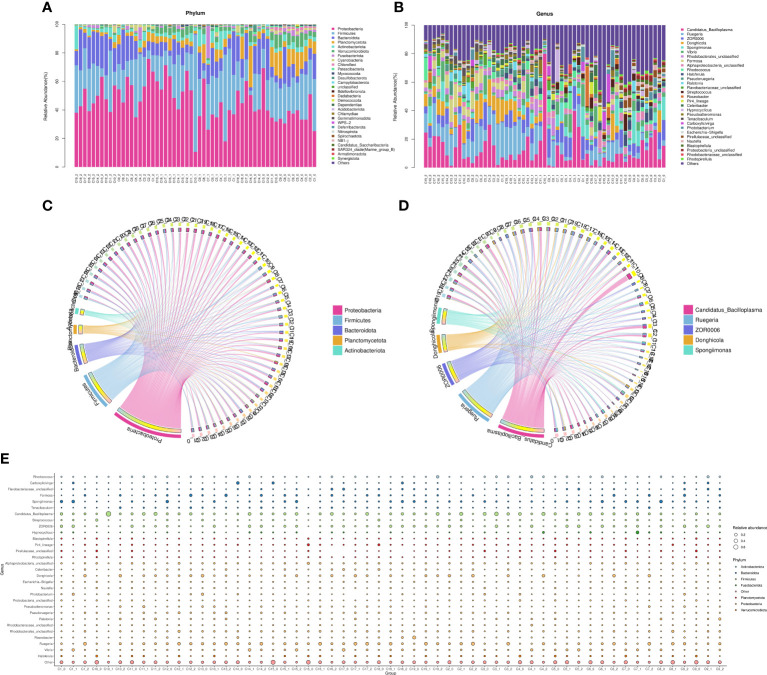
The microbial community composition in the intestine. **(A)** The distribution of the top 30 relative abundant species in each group at phylum level, **(B)** The distribution of the top 30 relative abundant species in each group at genus level, **(C)** The circus of top 5 relative abundant species in each group at phylum level, **(D)** The circus of top 5 relative abundant species in each group at genus level, **(E)** Species annotation information and relative abundance (circle size) at genus level in different sample groups.

**Figure 6:**
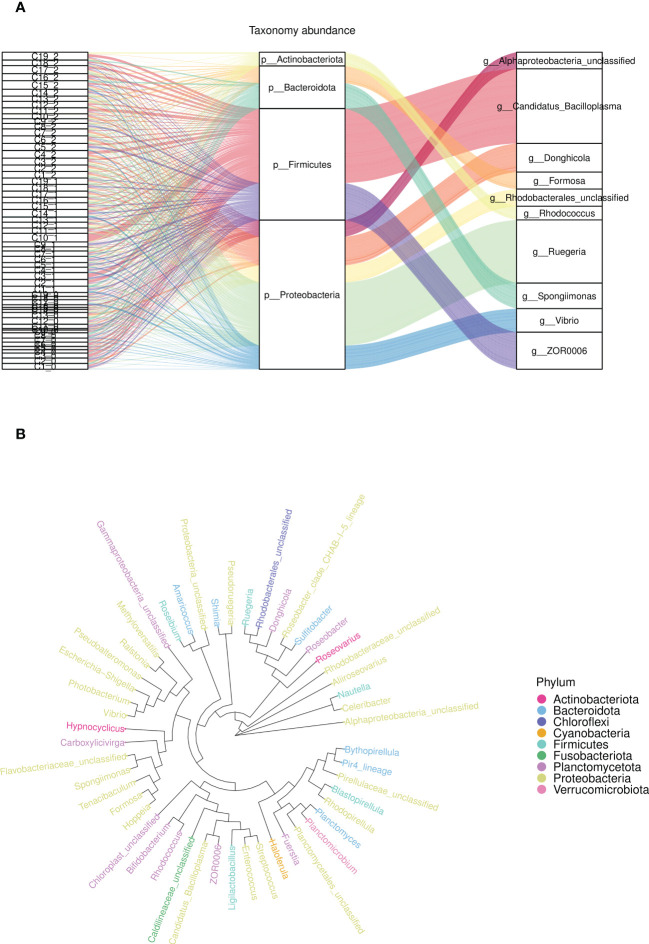
The relative abundance an phylogenetic tree of microbial community composition in the intestine. **(A)** The relative abundance of flora at phylum level (middle) and genus level (right) for different samples (left), **(B)** The phylogenetic tree of top 30 relative abundance species in each group at phylum level.

At the phylum level, the average intestinal microbiota community was dominated by *Proteobacteria*, *Firmicutes*, *Planctomycetes*, *Actinobacteria*, *Bacteroidetes*, *Camplylobacterota* and *Verrucomicrobia* (**Figure 5A**). Compared with the control groups, the relative abundances of *Proteobacteria*, *Bacteroidetes*, *Planctomycetes* and *Firmicutes* were significantly changed. Furthermore, there were more different phyla in the experimental groups (C1, C2, C4, C6, C7, C8, C9 and C15) than in the control groups, such as *Nitrospirae*, *Gemmatimonadetes* and *Bedllovibrionota.* At the genus level, the top 10 dominate species of the experimental group were *Enterorhabdu*, *Candidatus Bacilloplasma*, *Vibrio*, *Caldilinea*, *Donghicola*, *flavobacterium*, *Ruegeria*, *Spongiimonas*, *Pir4_lineage* and *Formosa* (**Figure 5B**). The relative abundance of *Vibrio* significantly decreased for C1, C2, C3, C4, C6, C7, C8, C9, C11, C13 and C15, while the relative abundances of *Bacteroidota* and *Donghicola* significantly increased (*p*<0.05) (**Figure 5E**). The distribution of the top five abundant intestinal flora of each sample at the phylum and the genus level, reflecting the composition proportion of dominant microbiological species in each group, and the distribution proportion of dominant microbiological species among different groups, were exhibited in **Figures 5C, D**, respectively. **Figure 6A** illustrates that the relative abundance of *Candidatus Bacilloplasma* and *ZOR0006* under *Firmicutes*, and *Vibrio* under *Proteobacteria* of C1, C2, C3, C4, C6, C7, C8, C9, C10, C11, C15 decreased, while the relative abundance of *Ruegeria* and *Alphaproteobacteria* under *Proteobacteria* increased. The phylogenetic tree at the genus level analysis indicated that bacteria belonging to *Proteobacteria* were most, followed by *Planctomycetota*, *Firmicutes*, *Bacteroidota*, and *Verrucomicrobiota*, etc. (**Figure 6B**).

### 3.7 Functional prediction of intestinal flora

**Figure 7:**
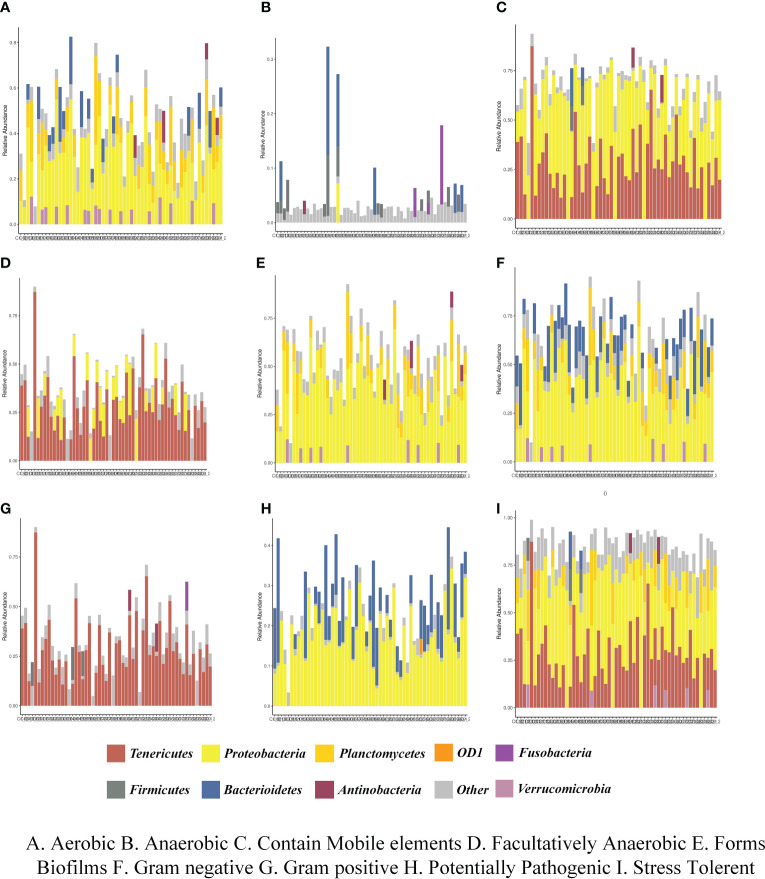
The relative abundances of species in phylum level with different phenotypes in different groups. **(A)** Aerobic, **(B)** Anaerobic, **(C)** Contain Mobile elements, **(D)** Facultatively Anaerobic, **(E)** Forms Biofilms, **(F)** Gram negative, **(G)** Gram positive, **(H)** Potentially Pathogenic, **(I)** Stress Tolerance.

Bugbase was applied to analyze the variety in the presumptive phenotypes of the intestinal microbiota of shrimps in each group after feeding. The main predicted phenotypes were aerobic, anaerobic, contains mobile elements, facultatively anaerobic, forms biofilms, Gram-negative, Gram-positive, potentially pathogenic, and stress tolerant. The relative abundances of species in phylum level with different phenotypes in different groups were shown in **Figure 7**. The results indicated that the phenotypes of aerobic, contains mobile elements, forms biofilms, Gram-negative, potentially pathogenic, and stress tolerant were closely relevant with *Proteobacteria* and *Tenericutes* in each group. PICRUSt2 was used to analyze the variety in the presumptive functions of the intestinal microbiota of shrimps in each group after feeding. The results of pairwise comparison between all experimental groups and control groups were shown in **Supplementary Figure S2**, indicating that the pathways of *Staphylococcus aureus* infection, degradation of ethylbenzene, amoebiasis infection, taurine and hypo taurine metabolism, antigen processing and presentation, and pentose and glucuronic acid conversion were significantly strengthened. Additionally, the pathways of cysteine and methionine metabolism, lysine biosynthesis, and ansamycin biosynthesis were significantly attenuated.

## 4 Discussion

The immunoprotected effects of CpG-ODNs have been reported in mice and other model organism. CpG-ODNs, as immunostimulatory agents, have been proven to stimulate immune cells and trigger immune responses to infections in many vertebral species, including humans (30), cattle and sheep (31), mice (32), poultry (33), and teleost fish (34, 35). There have also been studies on CpG-ODNs enhancing innate immunity in invertebrates, inducing autophagy through reactive oxygen species (ROS) in *Eriocheir sinensis* (36), enhancing hemolytic immune responses of *Macrobrachium rosenbergii* (37), and triggering hemocytes immune responses in *Litopenaeus vannamei* (38). In this study, different kinds of CpG ODNs produce discrepant immune-stimulated effects on account of their sequence divergence. Specifically, the expression of innate immune-related genes increased, the antioxidant capacity in hepatopancreas enhanced and the structure of intestinal flora improved.

The expression profiles of immune-related genes, including the *IL-1β*, *IL-8*, *IL-10*, *TNF-α*, *ALF* and *TOR* of *L. vannamei* fed with CpG 1826, CpG 1681, CpG 2006, CpG 8954, CpG M362, CpG 1585, and CpG 2143 exhibited significant upregulation. An interleukin (*IL*) system is essential for the proper regulation of T cell proliferation accompanied with an antigen encounter, as well as the modulation of apoptosis (39). Additionally, the *IL* family was well documented in invertebrates for its crucial roles in regulating innate immune system, such as in Chinese mitten crab (40) and in *L. vannamei* (41). The high expression of *IL-1β*, *IL-8* and *IL-10* stimulated by CpG ODNs indicated that the innate immunity of shrimps was effectively improved. *TOR* is highly conserved in eukaryotic organisms, and can integrate various biological processes such as translation, metabolism and autophagy of nutrient and growth factor regulatory proteins, and play a core regulatory role in cell proliferation, growth and metabolism (42). Autophagy has been demonstrated to play a pivotal function in shrimp innate immunity against invading pathogens (43). Similarly, there were significantly inductive effects for immune-related factors from CpG ODNs, including inflammatory cytokines, and anti-lipopolysaccharide factor, such as CpG 1681 and CpG 2006 in the spleen of grass carp *Ctenopharyngodon idella* (44), CpG M362 in murine plasmacytoid dendritic cells (45), CpG 1585 in serum of olive flounder (*Paralichthys olivaceus*) (46), CpG 8954 in pigs (47) and CpG 2143 in silver catfish (*Rhamdia quelen*) (48). The high expression of *TNF-α* and *ALF* in this study were consistent with the previously mentioned studies, which suggested that the immunity of shrimps was significantly enhanced as a result of orally administered CpG ODNs.

The increase in the antioxidant capacity in the hepatopancreas of *L. vannamei* illustrated that partial CpG ODNs could not only work at the genic level, but could also affect downstream protein expression. The antioxidant enzymes activity, including GST, CAT, GPX and SOD, plays a significant role in the innate immune system due to the absence of acquired immunity in *L. vannamei*. GST and GPX are important detoxification enzymes involved in apoptosis, cell proliferation, drug resistance and stress response (49). CAT and SOD are considered vital to survival because they eliminate ROS after pathogen invasion in the intestine (50). Early research only investigated the roles of CpG ODNs in hemocytes of shrimps, without a comprehensive or detailed evaluation (38). However, the positive effects on the antioxidant enzymes, together with immune-related genes in this study, proved that CpG ODNs could significantly enhance the innate immunity of *L. vannamei* administered orally.

The shrimp hepatopancreas comprises many tubules, mainly consists of basement membrane, cell layer, and hepatic canaliculus, and form the basic structural and functional units for absorption and digestion (51). The effective CpG-ODNs group in the study showed tight arrangement of tubules, complete basement membrane structure, and regular lumen shape, which improved the antioxidant properties of hepatopancreas, led to the repair of hepatopancreas damage, and maintained normal tissue and function. However, the increase in some inflammatory factors may also lead to corresponding histomorphological changes. The serious collapse and vacuolization of the hepatopancreas might be due to the dramatic increase in *TNF-α* after continuous CpG ODNs feeding, indicating that the immune response was strengthened.

The intestinal microbiota could participate in the regulation of multiple stress reactions, including high temperature, high pressure and other adverse conditions, by interacting with the host immune and digestive system (52). The roles of intestinal flora in the immunological stress of aquatic organisms have been widely studied, such as in *Trachinotus ovatus* and *Scophthalmus maximus* (53). CpG ODNs, which are in possession of similar bacterial DNA motifs, can be recognized by immune-related cells, such as phagocytes. However, there are fewer studies exploring the effects of CpG ODNs on the intestinal microbiota of aquatic organisms such as *L. vannamei*. In this study, there was strong evidence to suggest that different types of oral CpG ODNs administration modulate the structure and diversity of intestinal microbiota in *L. vannamei*, enhancing its innate immunity. The results indicated that the quantity and diversity of intestinal microorganisms significantly increased in partial CpG ODNs groups compared to the negative control groups, which was consistent with the regulation functions of the intestinal microbiota flora in terms of the nutritional composition changes or feed additives (54). In addition, the differentiate feature in the changes of the intestinal microbiota, both at the phylum and genus levels, of the effective dietary CpG ODNs groups was that pathogenic microorganisms decreased significantly, while beneficial microorganisms increased significantly. The dominant bacteria phylum were *Proteobacteria*, *Firmicutes*, *Planctomycetes*, *Actinobacteria*, and *Bacteroidetes*, which is consistent with previous results in fish and shrimp (54, 55). Furthermore, the relative abundances of *Nitrospirae*, *Gemmatimonadetes* and *Bedllovibrionota* were increased by the addition of partial CpG ODNs, supporting the point that the intestinal flora of shrimps became multitudinous. The variation tendency of microbial community at the genus level was consistent with those at the phylum level. *Vibrio* has been characterized as the dominant pathogenic bacterium in aquatic animals, and it can cause lethal diseases in shrimp (20, 22, 56). The relative abundance of the dominant genus *Vibrio* was significantly decreased in partial CpG ODNs groups in this study.

The results of functional annotation of key intestinal flora indicated that several pathways involved in immunity were strengthened, such as *Staphylococcus aureus* infection, amoebiasis infection and antigen processing and presentation. These innate immune responses were typically initiated by the recognition of PAMPs *via* PRRs due to the peculiar motifs of CpG ODNs. This indicated that CpG ONDs initiated the immunity of shrimps by activating related pathways and then improving other indicators. In addition, no studies have shown adverse effects from low concentrations of CpG ODN, which was also proved by the results of the hepatopancreas histomorphology of the treated shrimps in this study.

## 5 Conclusion

The present study showed that several dietary CpG ODNs can improve the intestinal health and immunity of shrimp without adverse effects, including CpG 2395, CpG 1585, CpG 1681, CpG 8954, CpG 2143, CpG 21424, CpG 21425, CpG 21426, CpG 2429, CpG 2216, and CpG M362. The enhancement covered immune-related genes, antioxidant enzymes and intestinal microbial diversity. These results indicated that the potential application of CpG ODNs improved the immunity of shrimp and laid a foundation for further investigation of its molecular series.

## Data availability statement

The data presented in the study are deposited in the NCBI repository, accession number PRJNA877472.

## Author contributions

FH: Writing – original draft, conducted the experiments and wrote the manuscript. YW: conducted the experiments, data curation, analyzed the data. JH: conceived the study and designed the experiments, checked, and modified the manuscript. ZB: conceived the study and designed the experiments, checked, and modified the manuscript. All authors read and approved the final manuscript. MW: conceived the study and designed the experiments, checked, and modified the manuscript. All authors contributed to the article and approved the submitted version.
