# Supplementary material for: Comparative study of the impact of dietary supplementation with different types of CpG oligodeoxynucleotides (CpG ODNs) on enhancing intestinal microbiota diversity, antioxidant capacity, and immune-related gene expression profiles in Pacific white shrimp (Litopenaeus vannamei)
Source: Front Immunol. 2023 Apr 27;14:1190590. doi: 10.3389/fimmu.2023.1190590 (PMC10174297; doi:10.3389/fimmu.2023.1190590)

Supplementary Material

**Comparative study of dietary supplementation with different types of CpG oligodeoxynucleotides (CpG ODNs) on enhancing intestinal microbiota diversity, antioxidant capacity, and immune-related genes expression in Pacific white shrimp (*Litopenaeus vannamei*)**

# Supplementary Figures and Tables

# 1.1 Supplementary Tables

Table S1 The type, sequence, and source information of the synthesized CpG ODNs.

| **Type** | **Name** | **Sequence (5’→3‘)** | **Reference** |
| --- | --- | --- | --- |
| CpG-A/CpG-D | 2216 | GGGGGACGATCGTCGGGGGG | (Kang and Kim, 2012; Strandskog et al., 2008) |
|  | 8954 | GgGGACGACGTCGTGgggggG | (Dar et al., 2010) |
|  | 1585 | ggGGTCAACGTTGAgggggg | (Martinson et al., 2007) |
| CpG-B/CpG-K | 2006 | TCGTCGTTTTGTCGTTTTGTCGTT |  |
|  | 1826 | tccatgacgttcctgacgtt | (Meng et al., 2003) |
|  |  |  | (Rhodes et al., 2004) |
|  | 1681 | ACCCGATGTCGTTGCCGGTGACG | (Jørgensen et al., 2001) |
|  | 2143 | TTCGTCGTTTTGTCGTTTTGTCGTT | (Carrington et al., 2004) |
| CpG -C | 2395 | TCGTCGTTTTCGGCGCGCGCCG | (Lin et al., 2015) |
|  |  |  | (Martinson et al., 2007) |
|  | M362 | TCGTCGTCGTTCGAACGACGTTGAT | (Li et al., 2011) |
|  | 2429 | TCGTCGTTTTCGGCGGCCGCCG | (Dar et al., 2010) |
| CpG -P | 23617 | TCGTCGACGATCGGCGCGCGCCG | (Samulowitz et al., 2010) |
|  | 21424 | TCGTCGTTACGTAACGTCGTCGTT | (Vollmer and Krieg, 2009) |
|  | 21425 | TCGTCGTTACGTAACGACGTCGTT |  |
|  | 21426 | TCGTCGTTACGTAACGACGACGTT |  |
| CpG-negative | 2137 | TGCTGCTTTTGTGCTTTTGTGCTT | (Jørgensen et al., 2001; Kang and Kim, 2012; Zhang et al., 2010) |
|  | 1720 | TCCATGAGCTTCCTGATGCT |  |
|  | 2243 | GGGGGAGCATGCTGGGGGGG |  |

Table S2 Naming rules for each group and sample in this experiment.

| Abbreviation | Name | Note |
| --- | --- | --- |
| C1 | CpG ODN 1826 | C_-0-1、C_-0-2、C_-0-3 represents three biological replicate samples of the same group taken at the first time (i.e., day 0)  C_-1-1、C_-1-2、C_-1-3 represents three biological replicate samples of the same group taken for the second time (i.e., day 7)  C_-2-1、C_-2-2、C_-2-3 represents three biological replicate samples of the same group taken for the second time (i.e., day 14) |
| C2 | CpG ODN 1681 |  |
| C3 | CpG ODN 2006 |  |
| C4 | CpG ODN M362 |  |
| C5 | CpG ODN 2137 |  |
| C6 | CpG ODN 8954 |  |
| C7 | CpG ODN 1585 |  |
| C8 | CpG ODN 2143 |  |
| C9 | CpG ODN 2395 |  |
| C10 | CpG ODN 2216 |  |
| C11 | CpG ODN 23617 |  |
| C12 | CpG ODN 2429 |  |
| C13 | CpG ODN 21424 |  |
| C14 | CpG ODN 21425 |  |
| C15 | CpG ODN 21426 |  |
| C16 | CpG ODN 2243 |  |
| C17 | CpG ODN 1720 |  |
| C18 | Negative control 1 |  |
| C19 | Negative control 2 |  |

Table S3 Microbial Alpha diversity of all samples

| Sample | Alpha diversity | | | | Coverage |
| --- | --- | --- | --- | --- | --- |
|  | Shannon | Simpson | Chao | Pielou_e |  |
| C1_0 | 4.9217±1.1853^b^ | 0.9068±0.05778^a^ | 304.9765±78.3497^a^ | 0.5968±0.1327^b^ | 0.9987±0.0004 |
| C1_1 | 5.3924±0.6628^a^ | 0.8955±0.0807^b^ | 487.1947±22.2884^a^ | 0.6039±0.0704^a^ | 0.9997±0.0001 |
| C1_2 | 5.2268±0.4274^a^ | 0.9327±0.0606^a^ | 731.8761±376.3011^d^ | 0.6507±0.1487^a^ | 0.9994±0.0003 |
| C2_0 | 5.9915±1.6141^b^ | 0.9008±0.0884^b^ | 410.3657±53.7646^a^ | 0.6360±0.1263^b^ | 0.9989±0.0008 |
| C2_1 | 6.1582±1.9313^a^ | 0.9106±0.0257^a^ | 391.7296±141.6752^b^ | 0.6098±0.0195^a^ | 0.9996±0.0002 |
| C2_2 | 5.6937±0.8892^c^ | 0.9139±0.0730^b^ | 710.8817±320.8226^c^ | 0.6554±0.0890^b^ | 0.9997±0.0003 |
| C3_0 | 4.5201±0.4097^b^ | 0.8397±0.1032^c^ | 384.5648±68.2040^a^ | 0.5647±0.1028^b^ | 0.9992±0.0006 |
| C3_1 | 5.3670±0.1150^a^ | 0.9361±0.0102^a^ | 302.4246±147.6919^c^ | 0.6272±0.0219^a^ | 0.9996±0.0001 |
| C3_2 | 6.4838±0.2315^a^ | 0.9615±0.0082^a^ | 672.9248±152.3485^b^ | 0.6936±0.0386^a^ | 0.9996±0.0001 |
| C4_0 | 4.3703±0.4811^a^ | 0.8565±0.0357^b^ | 342.7710±110.5981^c^ | 0.5222±0.0287^a^ | 0.9990±0.0002 |
| C4_1 | 5.2515±2.0589^d^ | 0.8943±0.0768^b^ | 529.0102±457.0189^d^ | 0.6052±0.1297^b^ | 0.9990±0.0007 |
| C4_2 | 6.8921±0.3169^b^ | 0.9705±0.0060^a^ | 781.8289±82.9626^a^ | 0.7184±0.0237^a^ | 0.9996±0.0002 |
| C5_0 | 4.8629±0.5409^c^ | 0.8915±0.0232^b^ | 403.3307±136.1992^b^ | 0.5650±0.0372^a^ | 0.9991±0.0005 |
| C5_1 | 6.1179±1.3053^b^ | 0.9292±0.0583^b^ | 660.8592±309.2707^c^ | 0.6590±0.0961^b^ | 0.9992±0.0009 |
| C5_2 | 6.5594±1.2657^a^ | 0.9699±0.0189^a^ | 628.1880±393.6700^d^ | 0.7287±0.0370^a^ | 0.9995±0.0002 |
| C6_0 | 5.0776±0.2343^a^ | 0.9699±0.0123^a^ | 343.8769±53.0640^a^ | 0.6046±0.0166^a^ | 0.9991±0.0003 |
| C6_1 | 6.0367±1.0121^b^ | 0.9397±0.0321^a^ | 622.9895±222.8649^c^ | 0.6538±0.0743^b^ | 0.9992±0.0006 |
| C6_2 | 7.1054±0.5646^a^ | 0.9241±0.0139^a^ | 801.8129±217.5938^c^ | 0.7407±0.0491^a^ | 0.9996±0.0001 |
| C7_0 | 5.2582±0.2140^a^ | 0.9378±0.0117^a^ | 385.3088±32.7403^a^ | 0.6139±0.0262^a^ | 0.9985±0.0004 |
| C7_1 | 5.0167±0.8709^b^ | 0.8922±0.0685^b^ | 390.6023±99.0560^b^ | 0.5852±0.0874^b^ | 0.9994±0.0003 |
| C7_2 | 6.7014±1.4890^c^ | 0.9467±0.0644^a^ | 822.1780±166.8947^b^ | 0.6933±0.1343^b^ | 0.9995±0.0001 |
| C8_0 | 4.9689±0.6890^c^ | 0.9100±0.0356^b^ | 282.2719±52.3091^b^ | 0.6125±0.0875^b^ | 0.9997±0.0001 |
| C8_1 | 4.8093±0.8836^b^ | 0.8901±0.0598^b^ | 346.8777±98.4862^b^ | 0.5720±0.0817^b^ | 0.9985±0.0002 |
| C8_2 | 7.1491±0.1205^a^ | 0.9679±0.0062^a^ | 877.8066±21.6208^a^ | 0.7331±0.0136^a^ | 0.9995±0.0003 |
| C9_0 | 5.3213±0.9422^c^ | 0.9296±0.0396^a^ | 295.2614±142.4242^c^ | 0.6611±0.0810^b^ | 0.9989±0.0001 |
| C9_1 | 5.5483±1.2132^d^ | 0.9271±0.0346^a^ | 403.2631±242.8099^d^ | 0.6494±0.0771^b^ | 0.9997±0.0007 |
| C9_2 | 7.3955±0.0738^a^ | 0.9815±0.0026^a^ | 870.5640±24.7919^a^ | 0.7585±0.0069^a^ | 0.9995±0.0002 |
| C10_0 | 2.8345±0.9200^d^ | 0.5652±0.1148^c^ | 330.0008±116.7425^c^ | 0.3266±0.0863^b^ | 0.9991±0.0005 |
| C10_1 | 5.1845±0.3275^b^ | 0.9140±0.0521^b^ | 410.1224±147.3966^c^ | 0.6288±0.0618^a^ | 0.9993±0.0001 |
| C10_2 | 7.2574±0.5346^a^ | 0.9745±0.0135^a^ | 821.9510±189.3393^b^ | 0.7518±0.0290^a^ | 0.9995±0.0001 |
| C11_0 | 4.3630±1.9503^c^ | 0.7654±0.3067^d^ | 260.4747±89.4166^c^ | 0.5411±0.2239^c^ | 0.9989±0.0007 |
| C11_1 | 5.4662±0.2887^a^ | 0.9234±0.0335^a^ | 395.5931±115.1318^d^ | 0.6397±0.0646^a^ | 0.9995±0.0003 |
| C11_2 | 6.9613±0.3080^b^ | 0.9643±0.0186^a^ | 744.4137±278.4139^c^ | 0.7382±0.0348^a^ | 0.9997±0.0002 |
| C12_0 | 5.0207±0.9422^b^ | 0.9023±0.0778^b^ | 350.0431±41.7294^a^ | 0.5943±0.1027^b^ | 0.9996±0.0001 |
| C12_1 | 5.3293±0.2580^a^ | 0.9535±0.0117^a^ | 505.5188±76.6540^a^ | 0.6715±0.0106^b^ | 0.9993±0.0002 |
| C12_2 | 6.0140±0.2234^a^ | 0.9313±0.0106^a^ | 307.1166±96.1617^c^ | 0.6496±0.0047^a^ | 0.9997±0.0001 |
| C13_0 | 5.0913±0.5489^c^ | 0.9010±0.0282^b^ | 394.4646±140.4784^b^ | 0.5945±0.0280^a^ | 0.9996±0.0002 |
| C13_1 | 5.7436±0.8109^c^ | 0.9443±0.0352^a^ | 339.7402±76.5508^b^ | 0.6575±0.0364^a^ | 0.9993±0.0003 |
| C13_2 | 5.2469±0.3411^b^ | 0.9248±0.0273^a^ | 447.3707±91.1438^a^ | 0.6291±0.0690^a^ | 0.9996±0.0004 |
| C14_0 | 4.5291±0.8523^c^ | 0.8770±0.0529^b^ | 273.3703±108.3383^d^ | 0.5624±0.0661^b^ | 0.9995±0.0004 |
| C14_1 | 4.5407±0.2158^a^ | 0.8766±0.0114^b^ | 302.1957±79.7115^b^ | 0.5550±0.0052^a^ | 0.9996±0.0001 |
| C14_2 | 5.4950±0.5099^b^ | 0.9367±0.0108^a^ | 460.7735±203.6303^d^ | 0.6260±0.0481^a^ | 0.9997±0.0001 |
| C15_0 | 4.7245±0.4171^b^ | 0.9162±0.0130^a^ | 279.2036±84.7264^b^ | 0.5852±0.0193^a^ | 0.9992±0.0004 |
| C15_1 | 5.42315±1.1213^d^ | 0.9375±0.0373^a^ | 432.7162±139.1339^c^ | 0.6211±0.0938^c^ | 0.9994±0.0008 |
| C15_2 | 5.7777±0.5940^c^ | 0.9462±0.0141^a^ | 523.2725±32.7201^a^ | 0.6416±0.0621^b^ | 0.9997±0.0006 |
| C16_0 | 4.6655±0.4076^b^ | 0.9067±0.0319^a^ | 245.3172±54.2587^b^ | 0.5908±0.0523^a^ | 0.9990±0.0008 |
| C16_1 | 6.6275±0.3231^a^ | 0.9603±0.0046^a^ | 778.8949±130.3032^b^ | 0.6916±0.0186^a^ | 0.9990±0.0002 |
| C16_2 | 7.8336±0.3563^a^ | 0.9890±0.0008^a^ | 799.9467±304.2770^c^ | 0.8215±0.0192^a^ | 0.9997±0.0001 |
| C17_0 | 4.8073±1.3918^c^ | 0.8496±0.1207^b^ | 395.3586±190.7684^c^ | 0.5618±0.1344^c^ | 0.9994±0.0004 |
| C17_1 | 5.2951±0.1631^a^ | 0.9320±0.0156^a^ | 355.4782±145.2912^c^ | 0.6365±0.0528^a^ | 0.9995±0.0003 |
| C17_2 | 5.1005±0.3974^b^ | 0.9186±0.0221^a^ | 409.3139±37.0280^a^ | 0.5890±0.0447^a^ | 0.9995±0.0002 |
| C18_0 | 6.4715±1.0551^b^ | 0.9653±0.0242^a^ | 609.3867±213.2322^b^ | 0.7034±0.0739^b^ | 0.9991±0.0004 |
| C18_1 | 5.6364±0.1281^a^ | 0.9427±0.0180^a^ | 463.0056±80.8513^a^ | 0.6394±0.0246^a^ | 0.9994±0.0001 |
| C18_2 | 4.7494±0.1148^a^ | 0.9078±0.0363^b^ | 332.5196±70.2780^b^ | 0.5708±0.0223^a^ | 0.9994±0.0005 |
| C19_0 | 4.5544±0.6457^b^ | 0.8616±0.0799^c^ | 345.0187±75.2839^b^ | 0.5423±0.0720^b^ | 0.9996±0.0001 |
| C19_1 | 5.8027±0.3710^a^ | 0.9467±0.0114^a^ | 500.1254±97.2736^a^ | 0.6495±0.0227^a^ | 0.9993±0.0003 |
| C19_2 | 4.7632±0.3143^b^ | 0.9030±0.0278^b^ | 306.6722±84.3249^b^ | 0.5834±0.0684^b^ | 0.99959±0.0002 |

The Shannon, Simpson, Chao, and Pielou-e indices of the samples were analyzed by SPSS 26.0 program for ANOVA and Duncan test for significance with *p* < 0.05, respectively.

# 1.2 Supplementary Figures


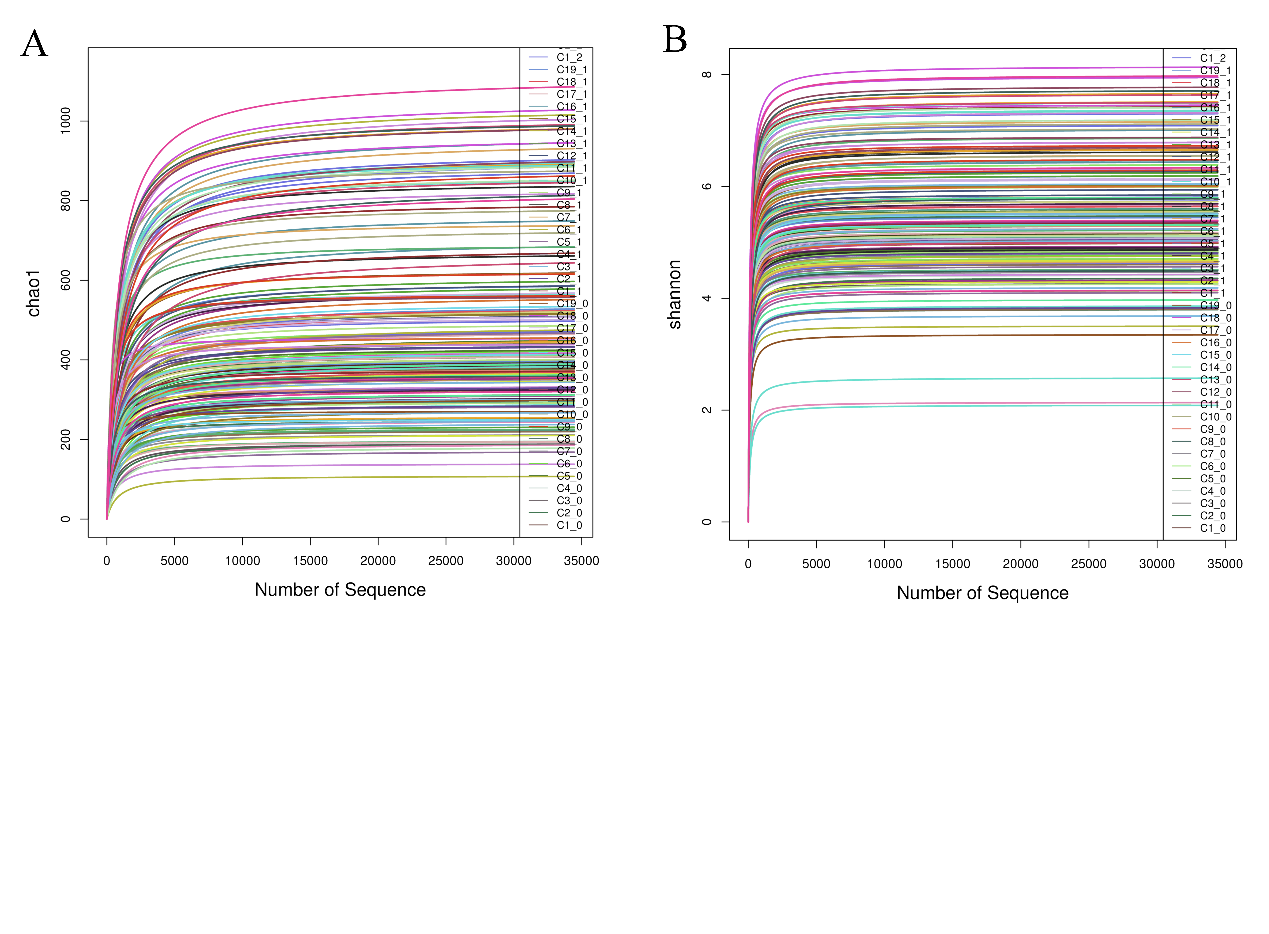


Fig. S1 Microbial Alpha diversity index curve of each sample. A. The rarefaction curve of chao1 of all samples; B. The rarefaction curve of Shannon of all samples.


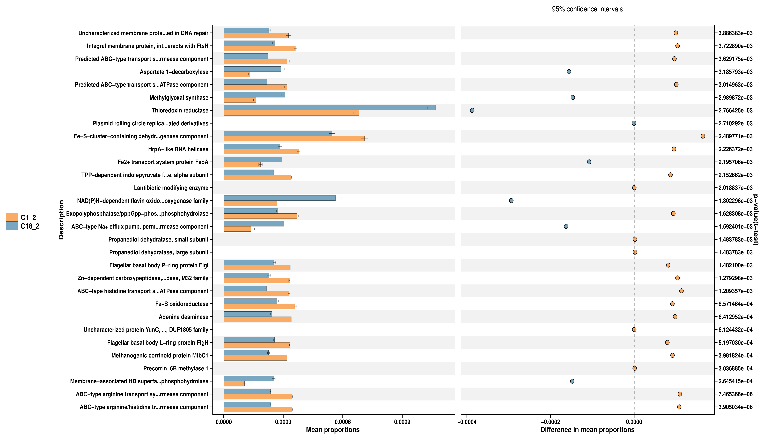

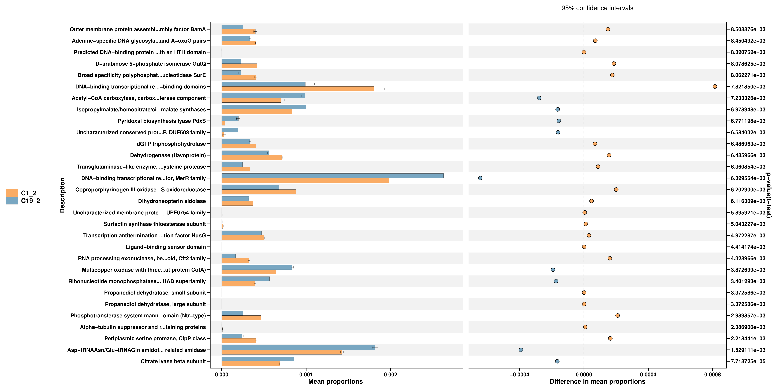


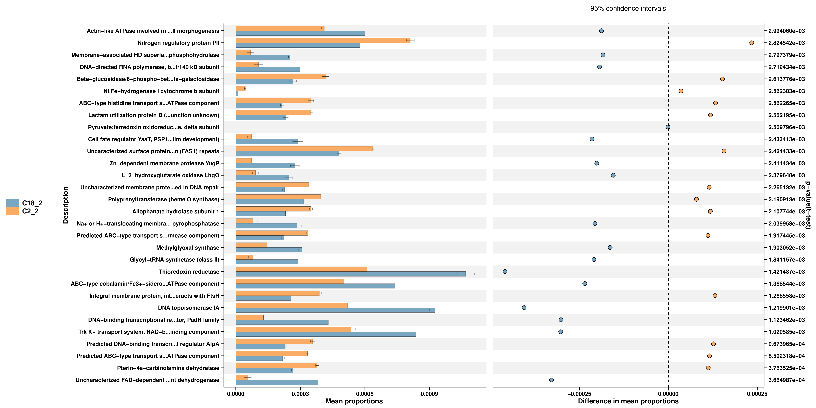


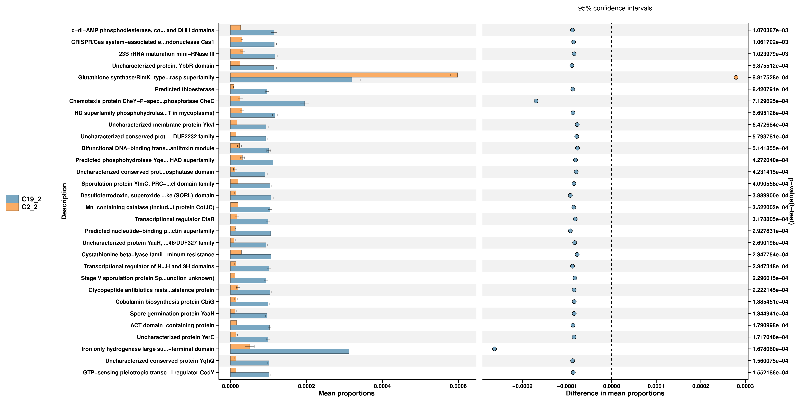


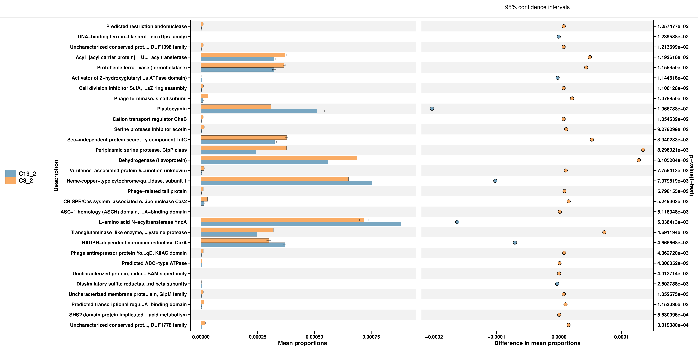

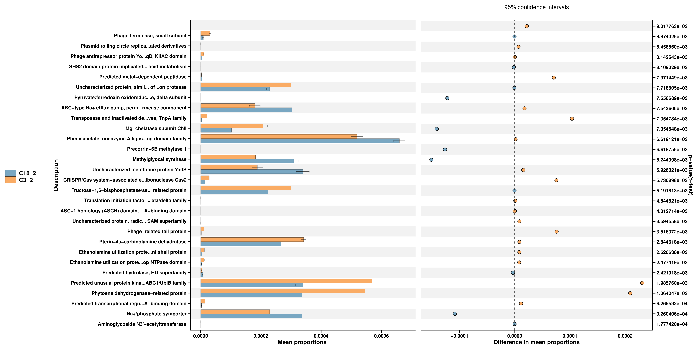


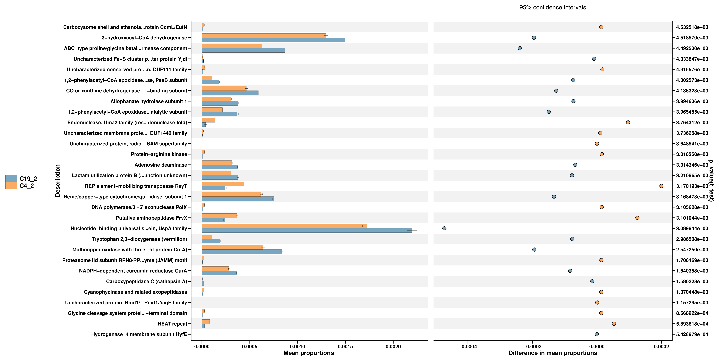

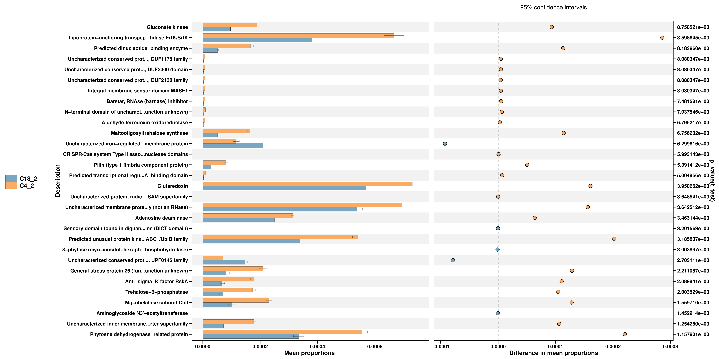


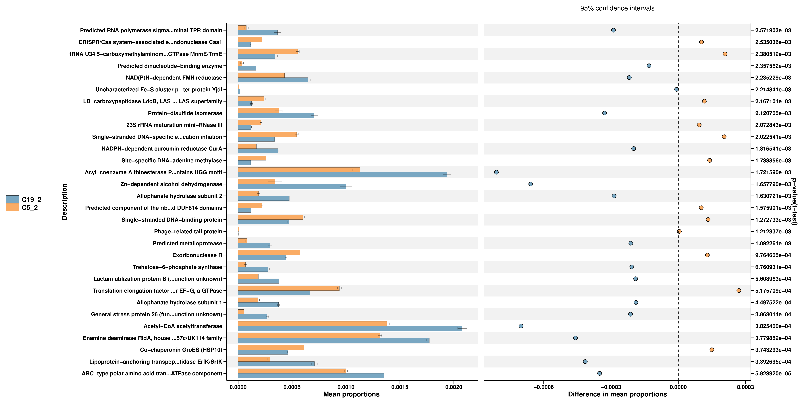

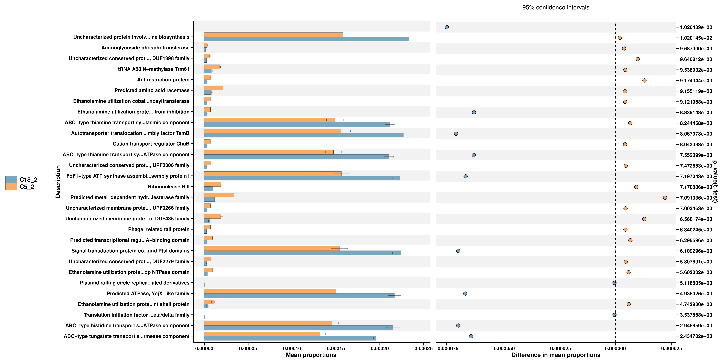


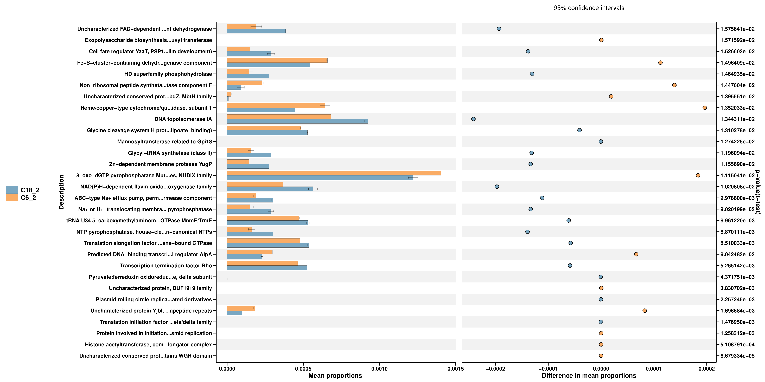


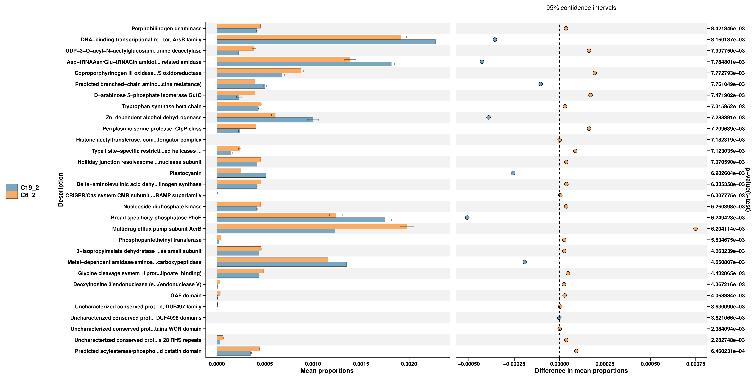


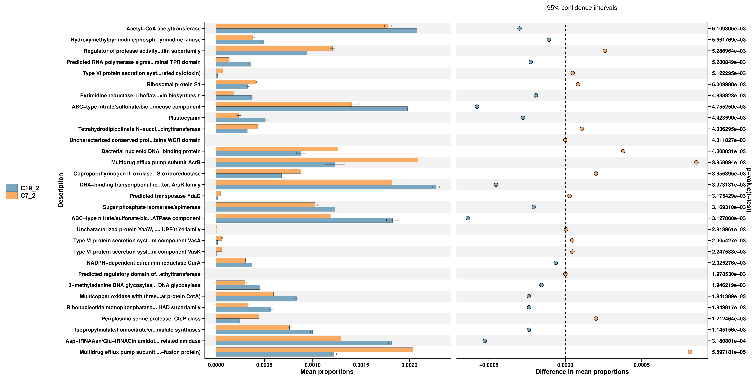

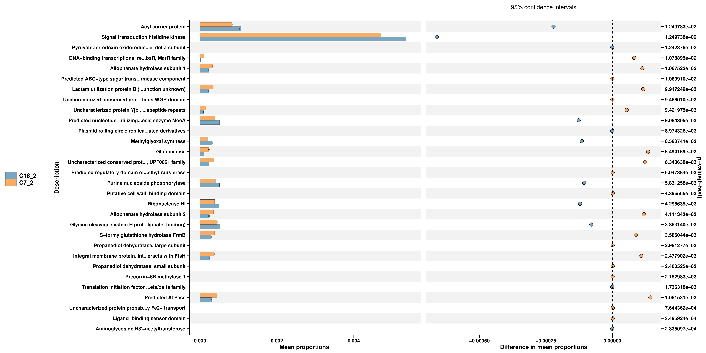


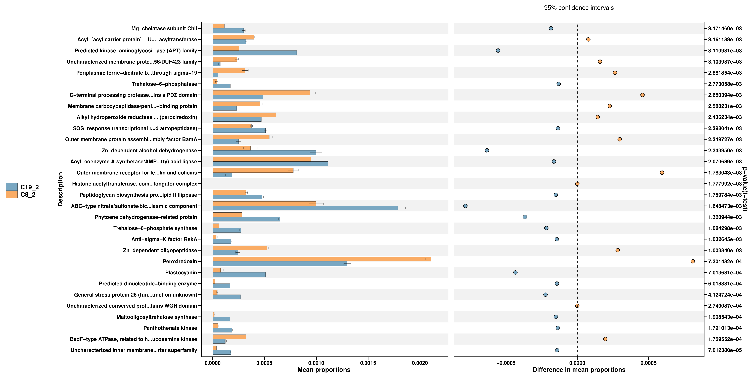


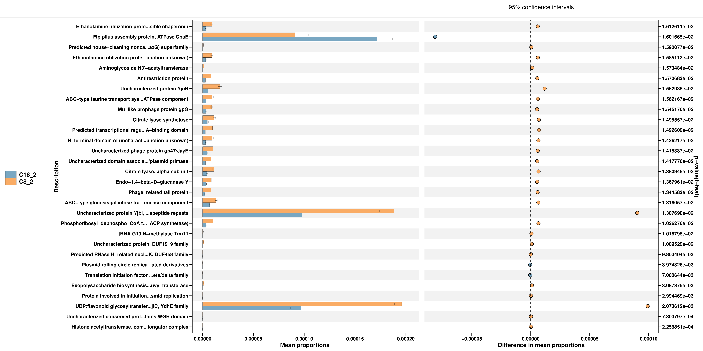


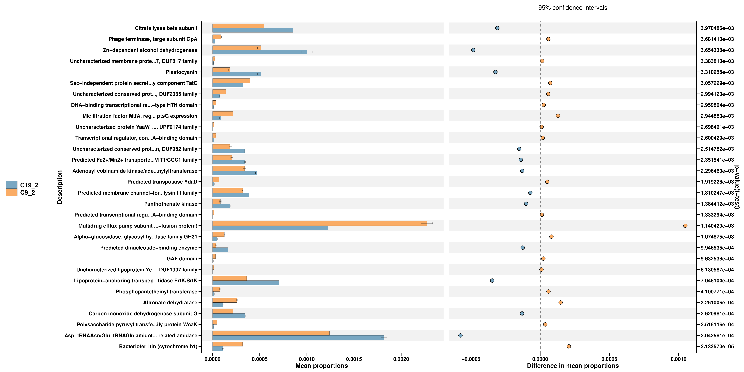

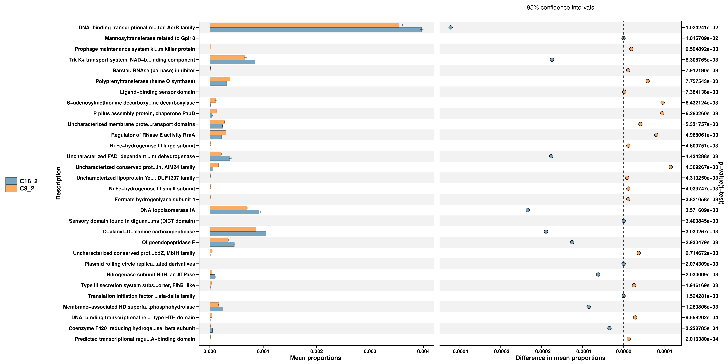


Fig. S2 Comparison of intestinal flora of *Litopenaeus vannamei* in healthy and disease groups with abundance of KEGG pathways related to disease, adaptation and immune pathways


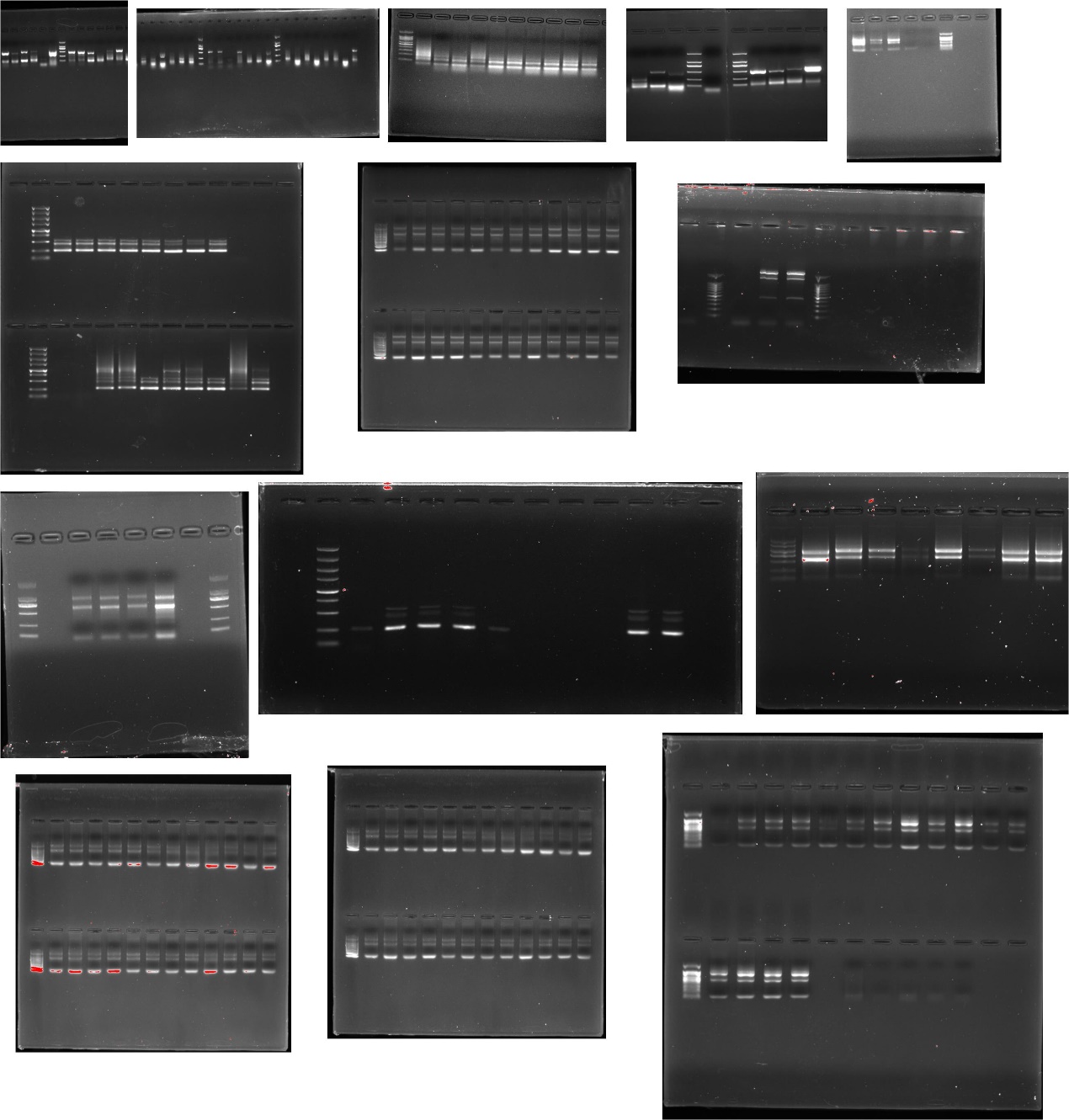

Supplement: Supplementary file 1 [file DataSheet_1.docx]
